# Sexual Conflict and Gender Gap Effects: Associations between Social Context and Sex on Rated Attractiveness and Economic Status

**DOI:** 10.1371/journal.pone.0146269

**Published:** 2016-01-05

**Authors:** Amany Gouda-Vossos, Barnaby J. Dixson, Robert C. Brooks

**Affiliations:** 1 Evolution & Ecology Research Centre, School of Biological, Earth & Environmental Sciences, University of New South Wales, Sydney, New South Wales, Australia; 2 School of Psychology, The University of Queensland, Brisbane, Queensland, Australia; Middlesex University London, UNITED KINGDOM

## Abstract

Human mate choice research often concerns sex differences in the importance of traits such as physical attractiveness and social status. A growing number of studies indicate that cues to social context, including other people who appear in stimulus photographs, can alter that individual’s attractiveness. Fewer studies, however, consider judgements of traits other than physical attractiveness, such as wealth. Here we manipulate the presence/absence of other people in photographs of target models, and test the effects on judgments of both attractiveness and earnings (a proxy for status). Participants (N = 2044) rated either male or female models for either physical attractiveness or social/economic status when presented alone, with same sex others or with opposite sex others. We collectively refer to this manipulation as ‘social context’. Male and female models received similar responses for physical attractiveness, but social context affected ratings of status differently for women and men. Males presented alongside other men received the highest status ratings while females presented alone were given the highest status ratings. Further, the status of females presented alongside a male was constrained by the rated status of that male. Our results suggests that high status may not directly lead to high attractiveness in men, but that status is more readily attributed to men than to women. This divide in status between the sexes is very clear when men and women are presented together, possibly reflecting one underlying mechanism of the modern day gender gap and sexist attitudes to women’s economic participation. This adds complexity to our understanding of the relationship between attractiveness, status, and sex in the light of parental investment theory, sexual conflict and economic theory.

## Introduction

According to evolutionary parental investment theory [1], females of most species invest more in each individual offspring and thus form a scarce resource, valuable to males as mates. As a result males tend to compete with one another for access to females and females choose the males best able to enhance their own, or their mutual offspring’s fitness. Parental investment theory (PIT) and the “males compete, females choose” (MCFC) paradigm it gave rise to has been superseded in recent years by more refined models [2, 3], but PIT and the MCFC paradigm still have a substantial hold on evolutionary thinking about human mate choice. A focus on PIT has led, however, to a very strong emphasis on sex differences in the kinds of traits each sex prefers.

Theories of mate value built on the foundation of PIT propose that traits that signal youth and fertility augment women’s physical attractiveness, whereas traits that signal status and dominance enhance male attractiveness [4, 5]. Men tend to prioritise cues of youthfulness in mates [6–8] and age-related physical cues such as facial shape, skin complexion, breast morphology and an hourglass distribution of body fat [9–14].

In contrast, masculine traits, such as muscularity, deeper vocal pitch, facial masculinity and beardedness may provide information relating to male age, health, social status physical strength, social dominance and formidability that may enhance male attractiveness [15–18]. In addition to physical formidability, social status measures such as achieved hierarchical rank or ascribed status are associated with greater mating success [16, 19–22].

Mate choice can also be substantially flexible, and individuals may be influenced by the mate choices of others [23]. Modelling of this kind of non-independent mate choice [24] suggests that the interactions between males and females are used by females to assess male mate quality. Experimental evidence demonstrates that the immediate social environment, such as the women a man is photographed alongside or seen to be interacting with, can influence female mate choice (either favourably or unfavourably), suggesting that social context plays a role in plasticity of mate preferences [25, 26].

A growing body of research has shown that social context causes plasticity in mate preferences. For example, wearing clothing indicating high social status [27, 28] and apparent ownership of luxury possessions [22, 29, 30] modifies judgements of attractiveness. These studies also reveal sex differences in judgements across varying social contexts, with cues pertaining to high status (e.g. luxury possessions) positively influencing judgements of attractiveness in men, but not in women. The presence and absence of others also influences ratings of attractiveness, so that men’s attractiveness is elevated when in the presence of a woman [31]. Such findings are often interpreted as ‘mate choice copying’, a phenomenon whereby an individual enjoys elevated chances of being chosen as a mate if others have previously mated with them [32]. Mate choice copying has been reported in fish [33, 34], birds [35, 36], and invertebrates [37].

In humans, mate choice copying may reflect social transmission of mate relevant information [23, 38]. Men photographed in the company of women receive elevated attractiveness ratings [31, 39, 40], reflecting that those men have desirable traits and/or are potentially non threatening. In contrast, a women’s attractiveness is negatively influenced by the presence of other men [31]. By preferring women who are currently or have been recently involved with other men, may, historically, have increased the man’s risk of misdirecting parental investment to the offspring of another male. This ‘desirability diminishing effect’ to females presented alongside a man [31] could also reflect mechanisms of intra-sexual competition wherein men’s attention is captured more by a potential rival who could present a physical threat than the female target of mate choice [41]. While religious or cultural practices may influence this effect, cross cultural studies have also shown that societies worldwide share some common attitudes towards sex and sexuality [42]. There are, however, some deviations. For example, chastity is more valued in China and India than in Sweden and Norway [6]; but within societies the value and importance of women’s chastity is still consistently higher compared to men.

Interestingly, when people are judged within non-mating pairs (i.e. same-sex friendships), an individual’s is only judged more favourably when presented alongside an attractive ‘friend’ [43]. Like mate choice copying, the social information about a friend can inform judgements of attractiveness of the target [44].

While contextual cues may influence sex differences in attractiveness judgments, many of these studies have focused on judgements of attractiveness without accounting for how changes in social context may influence other mate relevant traits, such as age, wealth and earning potential, as most experiments assessing mate relevant traits control for age.

In the current study, we tested the effects of an experimental ‘social context’ manipulation on ratings of male and female attractiveness and social status (using earnings as a proxy). This ‘social context’ manipulation involved presenting each model either alone, with a same-sex ‘other’, or with an opposite-sex ‘other’. Our analysis is presented in two parts. First, we report associations between judgements of attractiveness, social status, and age in the models when they were presented alone (i.e. outside of variation in social context). Based on ideas derived from parental investment theory [1, 5, 6, 8], we predicted that male attractiveness would be positively associated with both social status and age. For female models, we predicted that attractiveness would decline with age while social status would increase with age due to accumulation of experience and wealth. Although, some studies have also uncovered a ‘beauty premium’ by which attractive people (especially females) are seen to be better negotiators [45], have higher confidence and oral skills [46], which ultimately leads to increased monetary reward [47, 48]. Thus, we include an alternate hypothesis where female’s social status may, like attractiveness, decrease with age.

In the second part of our analyses, we assessed the influence of social context on judgements of attractiveness and social status. Based on studies of human mate choice copying [31, 49] and social transfer of mate preferences [38, 43, 44], we predicted that males would be more attractive when paired with a female than when alone or with other males, whereas female attractiveness would decrease when shown in the presence of a male. We also tested the prediction that male social status would increase with the presence of a female, whereas women’s social status would not. We then performed a secondary analysis to test if experimental results were modified by the age of the models. We predicted that older males presented alongside females would obtain the highest ratings of attractiveness and social status than their younger male counterparts.

## Materials and Methods

### 2.1 Stimuli

Colour photographs of 20 male and 20 female models were obtained from a stock photo website (www.peopleimages.com). All photographs were taken using standardised lighting and filters. Representatives from *People Images* assisted in finding photographs of each model in 3 social contexts; alone, with a same sex other and an opposite sex other. A total of 17 male and 15 female models had photographs in all of the 3 social contexts. All 15 of the female models were paired (in the opposite-sex treatment) to a male who was also in the male experiments. *PeopleImages*.*com* collects information on the models they recruit. This information includes biological sex, age and ethnicity. The models ages ranged from 22–61 years (males: mean = 40 ±12.3, females = 38 ±12.7) and the majority were classified as Caucasian (66%) followed by multi-ethnic (16%), then African and Latino (9% each).

### 2.2 Procedure

Experiments were conducted online (www.socialsci.com). Participants were recruited via a single entry point (URL), and then randomly assigned to one of four experiments: assessing male attractiveness (Experiment 1), female attractiveness (Experiment 2), male social status (Experiment 3) or female social status (Experiment 4).

Within each experiment, each model was shown once, in random order, without replacement. Each model was presented in only one of three social contexts, again at random. This permits a within-model comparison of the effects of the three social contexts.

Participants were provided with a screen of instructions for how to perform the ratings. They were then informed that they would see a range of images of people within which the model to be rated would be identified with an arrow. Participants were then instructed to rate the model using the scale provided at the bottom of each screen. Participants rated the attractiveness of each model using a sliding scale from 0–100 where ‘50’ indicated that the individual is more attractive than 50% of other individuals of the same sex (i.e. of average attractiveness). Participants rating status were asked to estimate the salary of each model using a sliding percentile scale from 0–100, where ‘50’ indicates the individual earns more than 50% of other same sex individuals in full time work (i.e. the average income in full time work).

### 2.3 Participants

Participants were recruited via social media outlets (i.e. Facebook and Twitter) and email lists of potential subjects (usually participants in our previous studies and people who have read news stories about our research) that we keep in our lab. Each participant provided their biological sex, age and sexual orientation using the Kinsey Scale [50] which is a 7 point heterosexual-homosexual scale which describes a person’s sexual orientation using a scale from 0 (exclusively heterosexual) to 6 (exclusively homosexual). Only participants who were over 18 and heterosexual or bisexual were retained in the analyses (i.e. Kinsey scale 0–3). Four hundred and thirteen men (aged 18–69; mean = 26.52 ±7.99) and 757 women (aged 18–57; mean = 26.42 ±7.30) rated model females while 131 men (aged 18–67; mean = 29.50 ±10.48) and 725 women (aged 18–61; mean = 27.06 ±7.21) rated model males. The majority of participants were from the United States of America (34.2%), followed by Australia (19.3%), United Kingdom (7.7%) and Canada and Germany (4.5% each). From this, 34.2% identified as North Western European, British or Irish, 17.7% European Mixed race and 7.4% South eastern and eastern Europe. It must be noted that a large portion (10%) stated that ‘none of these define my group/ or would rather not say. The demographic information we gathered from the participants is quite similar to what we know about the models. The majority of participants and models identify as Caucasian background with the second largest group identifying themselves as ‘mixed raced’. This is most likely because their county of residences are multicultural hubs, the chance of intermarriage is higher than in more homogenous places.

The majority of participants held an undergraduate degree (46%), followed by high school certificate/diploma (18.6%) and Post-Graduate Degree (17%). Before participants gave their consent, they were presented with an ‘information statement’, explaining the basic purpose of the study and what it entails. This information statement can be found in S1 File. As this was an online study, participants had to click an “I Agree” icon to continue. Participants were informed that by clicking “I Agree”, they were consenting to participate in the study. If they chose not to participate they were told that they could close the window by clicking the ‘x’ on the top right corner of the screen. This research and consent procedure was approved by the University of New South Wales Human Research Ethics Advisory Board (Psychology) (HREAP 1880).

## Results

### 3.1 Associations between attractiveness, social status and age

We first explored the relationships between the models age, rated attractiveness and social status, using only the aggregate attractiveness and social status ratings for each model in the ‘alone’ condition.

#### 3.1.1 Male models

Male model attractiveness ratings were negatively correlated with age (r = -0.676, *df* = 17, *P* = 0.003) and social status ratings (r = -0.487, *df* = 17, *P* = 0.047). Social status ratings were positively correlated with age (r = 0.768, *df* = 17, *P* < 0.001). Partial correlation analysis showed that attractiveness and age remained negatively correlated, even after accounting for the effects of social status (Partial r = -0.540, *df* = 14, *P* = 0.031). Likewise, social status ratings and age remained positively correlated after accounting for the partial effects of attractiveness (Partial r = 0.682, *df* = 14, *P* = 0.004). The correlation between attractiveness and social status ratings were no longer significant after controlling for age (Partial r = 0.068, *df* = 14, *P* = 0.802). Thus, older males were rated as less attractive and, independently, of higher social status compared to younger males.

#### 3.1.2 Female models

Female attractiveness ratings were also negatively correlated with the models age (r = -0.783, *df* = 13, P = 0.001), but not with social status ratings (r = 0.239, *df* = 13, *P* = 0.392). Social status ratings were also not significantly correlated with age (r = 0.371, *df* = 13, *P* = 0.174). Partial correlation analysis showed that attractiveness ratings and age remained negatively correlated after accounting for ratings of social status (Partial r = -0.770, *df* = 12, *P* = 0.001). Correlations between attractiveness and social status ratings remained non-significant after the effects of age were controlled (Partial r = 0.090, *df* = 12, *P* = 0.760). Thus, older females were rated as less attractive than younger females, but age did not co-vary with female rated social status, and nor did rated social status co-vary with attractiveness ratings.

### 3.2 Statistical analyses: Mixed Linear Model (MLM)

We analysed the results of each of the four experiments using a series of MLMs, where model ID was a repeated-measures factor, participant ID was a random factor, and social context and participant sex were fixed factors. We tested the main effects and the interactions between fixed factors. Restricted Maximum Likelihood (REML) methods were implemented when fitting our linear mixed models.

A subsequent series of MLMs were built to test the effects of models age and age^2^ as linear and quadratic covariates, as well as interactions between the fixed factors and these covariate terms. We built MLMs by fitting the random, repeated measures and fixed factor terms, including the interactions between the fixed factors. We then fitted the effects of the covariates via both forward addition and backward removal of covariates and covariate x fixed factor interactions. We present only the results from the final model that included the statistically significant terms following the forward-backward fitting process. All other MLMs that were constructed for attractiveness and social status ratings are provided in supplementary materials (S1 Table and S2 Table respectively).

#### 3.2.1 Effects of social contexts, sex and age on model male attractiveness

**Fig 1:**
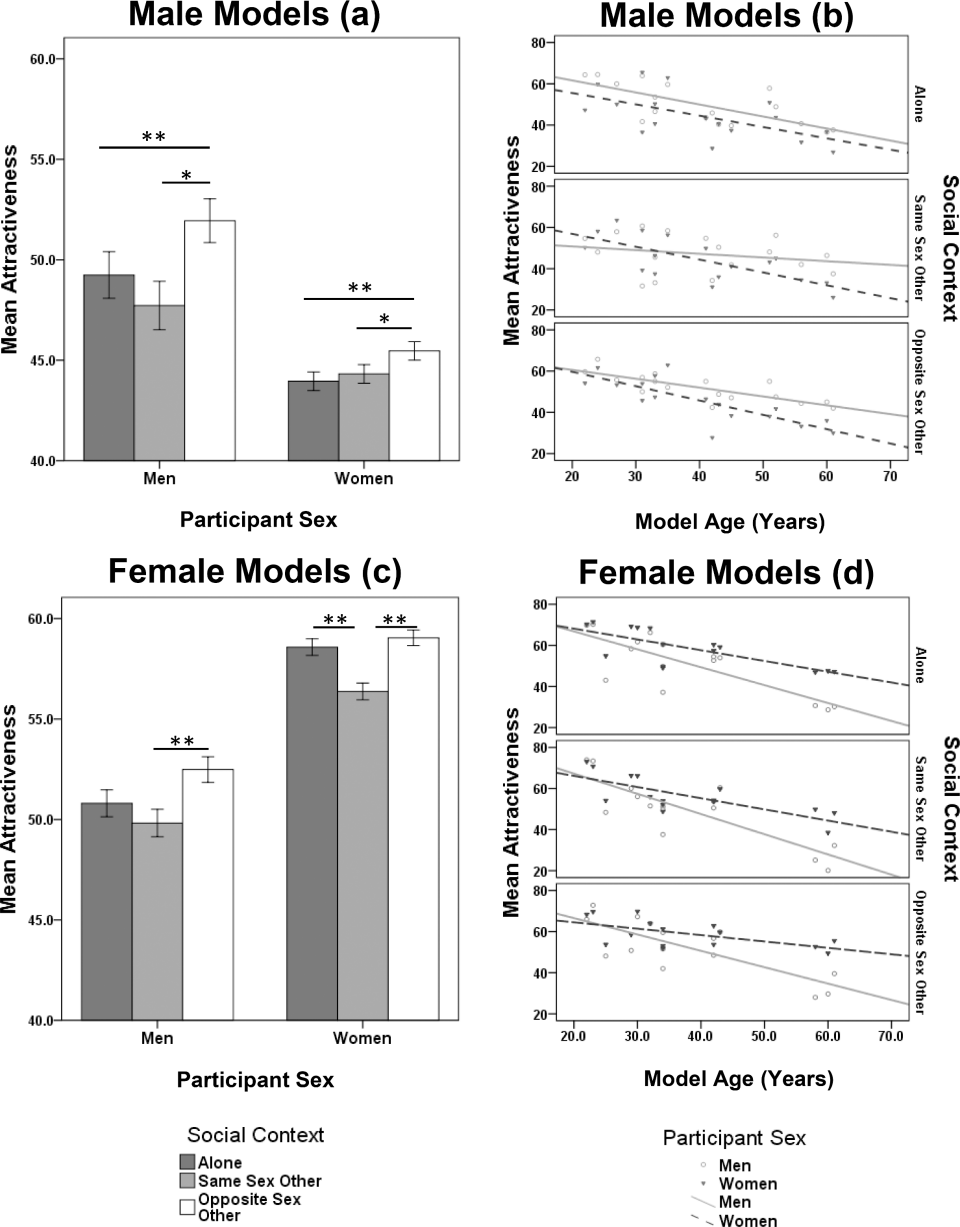
Effects of social contexts, sex and age on model male and female attractiveness. Data are mean attractiveness ratings (±1 SEM) for male (A) and female (C) targets split by sex of participant for 3 social contexts and attractiveness for male (B) and female (D) targets split by age and sex of participants. Lines within panels B and D indicate the effect of target age (in years) on rated attractiveness. *p <0.01, ** p <0.001 determined by post-hoc least significance difference tests.

Males received higher ratings when presented with an opposite sex other than when presented alone or with a same sex other (main effect of social context *F*_2,6605_ = 6.043, *P* = 0.002; Fig 1A). Both sexes rated males as more attractive when with opposite sex others. While there was a significant main effect of participant sex on attractiveness ratings (*F*_1, 6643_ = 47.669, *P* < 0.001), with men giving higher ratings than women, the social context × participant sex interaction was not statistically significant (*F*_2, 6605_ = 1.430, *P* = 0.239).

Consistent with the partial correlation analysis, attractiveness ratings decreased as model age increased (Fig 1B; *β* = -0.438 ± 0.0978 S.E.). Women’s ratings, however, appeared to be lower and slightly steeper compared to the ratings of men which was consistent with a participant sex x model age interaction (*β* = -0.260 ± 0.104 S.E.; *F*_1, 3605_ = 14.375, *P* <0.001) and thus driving a complex social context × participant sex × model age interaction (*F*_2, 3590_ = 3.894, *P* = 0.020).

#### 3.2.2 Effects of social contexts, sex and age on model female attractiveness

We found significant main effects of both social context (*F*_2, 8619_ = 15.968, *P* <0.001) and participant sex (*F*_1, 8628_ = 197.735, *P* <0.001) on ratings of female attractiveness. Overall, women gave higher attractiveness ratings to females than men but females presented alongside same sex others were rated as the least attractive by both men and women (Fig 1C). This was reflected in the non-significant social context × participant sex interaction (*F*_2, 8619_ = 1.174, *P* = 0.309).

When age was added as a covariate into the MLM, ratings of model females declined as age increased (Fig 1D; *β* = - 0.793 ± 0.0420 S.E.), which was consistent with the partial correlation analysis. However, men’s attractiveness ratings declined more steeply as the curve for women’s ratings was shallower than men’s (Fig 1D; *β* = 0.475 ± 0.053 S.E.). Overall, this trend was consistent across participant sex, so that the social context × participant sex × age interaction were not statistically significant (*F*_1, 4108_ = 1.766, *P* = 0.171).

#### 3.2.3 Effects of social contexts, age and sex on model male social status

**Fig 2:**
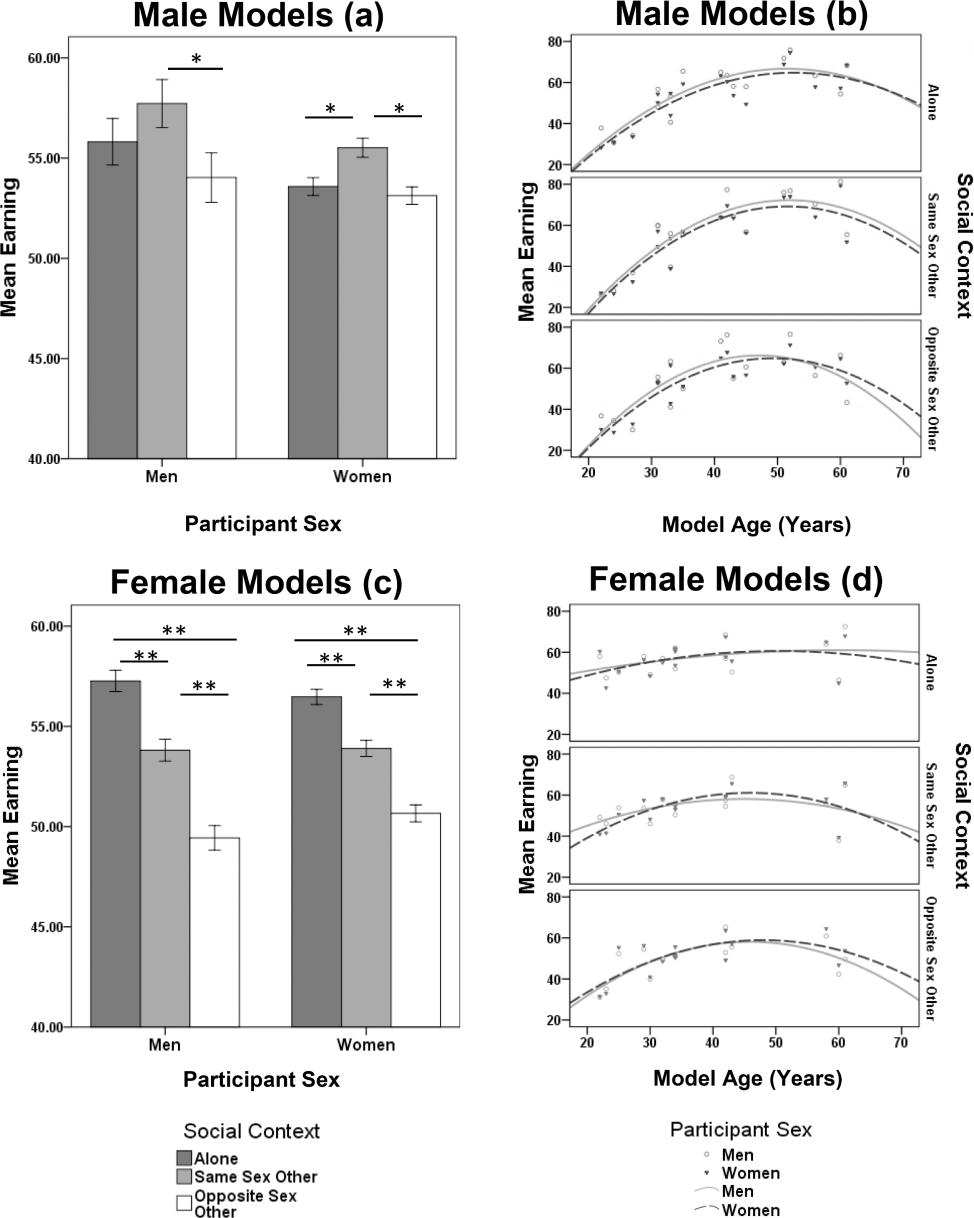
Effects of social contexts, age and sex on model male and female social status. Mean social status ratings (±1 SEM) for Male (A) and female (C) targets split by sex of participant for 3 social contexts and social status ratings of male (B) and female (D) models split by age and sex of participants. Lines within panels B and D indicate the effect of target age (in years) on rated earnings. *p <0.01, ** *p* <0.001 determined by post-hoc least significance difference (LSD) tests.

Males presented with a same sex other were given the highest ratings of earnings than when presented alone or with an opposite sex other (Fig 2A), which was reflected by a significant main effect of social context (*F*_2,5473_ = 9.000, *P* <0.001). There was also a significant main effect of participant sex (*F*_1, 5529_ = 7.825, *P* = 0.005), so that men gave higher ratings than women.

Model age exerted both linear (*β* = 5.117 ± 0.218 S.E.; *F*_1, 2745_ = 1439.191, *P* <0.001) and quadratic (*ɣ* = - 0.0526 ± 0.002; *F*_1, 3030_ = 980.531, *P* <0.001) effects on ratings of male social status. It appears that irrespective of social context and participant’s sex, ratings of male social status peaked at approximately 45–50 years old and declined thereafter (Fig 2B); however there were significant social context × model age (*F*_2, 2745_ = 5.456, *P* = 0.004) and social context × model age^2^ (*F*_2, 3028_ = 5.054, *P* = 0.006) interactions (Fig 2B). This may be due to participants rating older males presented alone slightly higher than when they were presented with an opposite sex other.

#### 3.2.4 Effects of social contexts, sex and age on model female social status

Female social status was rated highest when females were presented alone and lowest when presented with an opposite sex other (Fig 2C), which drove a significant main effect of social context on female social status ratings (*F*_2,7972_ = 80.182, *P* <0.001). There was no significant main effect of participant sex (*F*_1, 7980_ = 0.966, *P* = 0.326) or any social context × sex interaction (*F*_2, 7972_ = 1.604, *P* = 0.201).

Female social status ratings reflected a more complex pattern of age dependence than among males (Fig 2D). There were significant linear (*β* = 2.153 ± 0.184 S.E.; *F*_1, 6103_ = 288.596, *P* <0.001) and quadratic (*ɣ* = 0.022 ± 0.002; *F*_1, 6307_ = 207.468, *P* <0.001) effects of model age, and significant social context × model age (*F*_2, 6099_ = 11.891, *P* <0.001) and social context × model age^2^ (*F*_2, 6302_ = 12.739, *P* <0.001) interactions. When presented alone, the relationships between age and rated social status were almost flat, but when presented alongside same sex or opposite sex others ratings of social status rose and peaked at 40–50 years and declined thereafter; similar to ratings of male social status (Fig 2D).

There were also significant social context × participant sex × model age (*F*_2, 6099_ = 5.173, *P* = 0.006) and model age^2^ (*F*_2, 6302_ = 5.069, *P* = 0.006) interactions. The differences in age dependence, social context and sex were not obvious from inspecting Fig 2D or the parameter estimates, but they suggest additional complexity in the assessment of female social status in relation to context.

**Fig 3:**
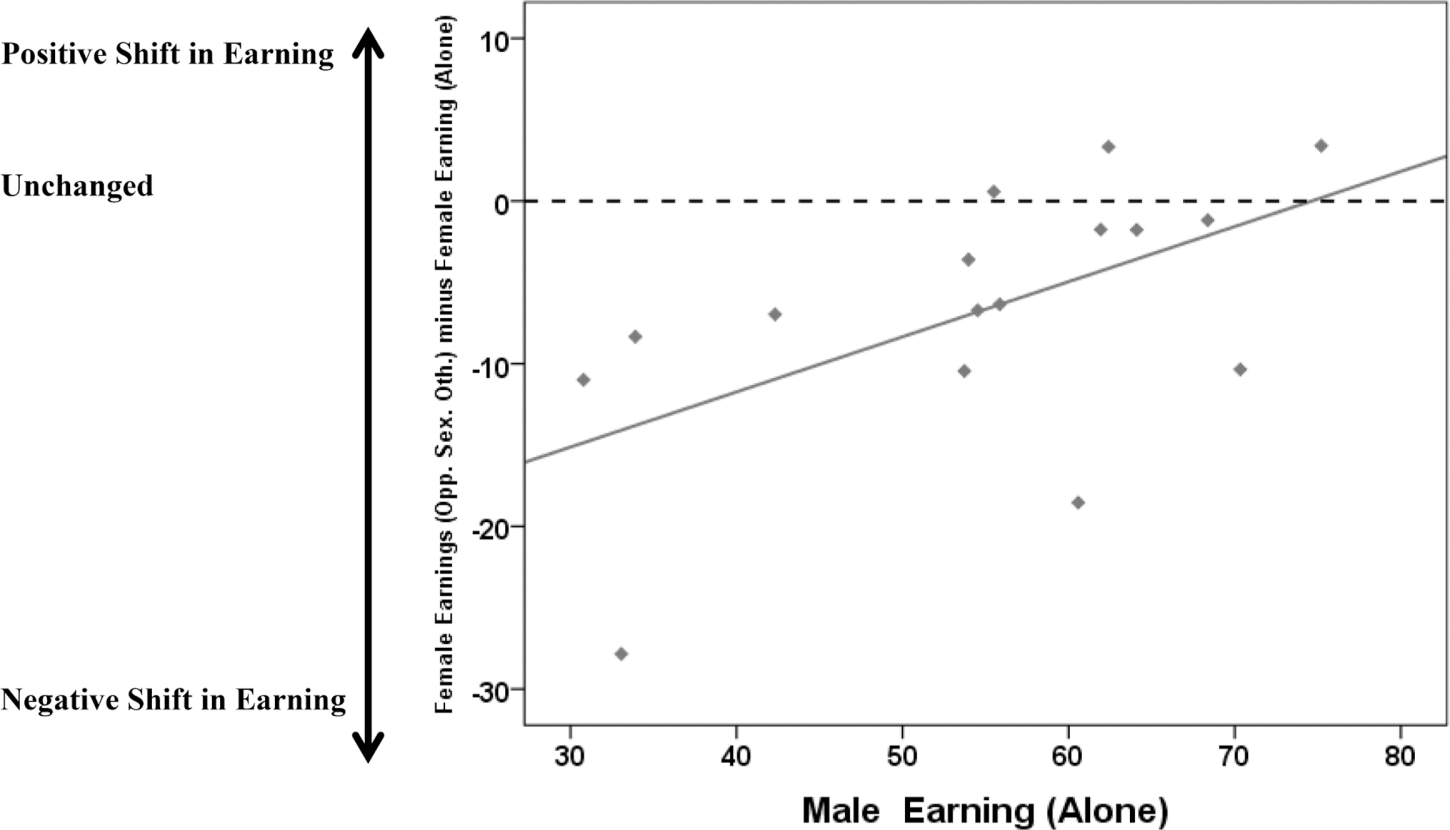
The influence of male social status on female social status. This figure shows the correlation (r = .564, n = 15, p = .028) between the shift in female rated social status when presented alone compared to when females were presented alongside a male (y-axis) plotted against male social status ratings when presented alone (x-axis). Female social status ratings were negatively impacted by the males they were presented alongside. The dotted line drawn at *y* = 0 indicates no shift in social status ratings.

An additional analysis exploring the influences of opposite sex others (males) on the ratings of female social status was conducted. We calculated the mean rated social status (separated by social context) for each of the male and the female models from the 15 male and female pairs that were used in both male and female experiments. We then calculated the difference in female social status between the ‘alone’ and ‘opposite sex other’ conditions, and tested whether this difference was correlated with the rated social status of the male when he was presented alone. There was a significant positive correlation (r = 0.564, n = 15, *P* = 0.028), reflecting that the decrease in female social status ratings when presented alongside a male was associated with his ‘alone’ social status ratings. Thus, females received their lowest social status ratings when presented alongside a male who had been given low social status ratings. Further, the overwhelming majority of model female’s social status shifted negatively when presented with a male (Fig 3), and only those women presented alongside men who appeared high-earning were unaffected by the presence of the male.

## Discussion

Social context altered how individuals in our experiment were rated for attractiveness and social status, but not always in the ways that we predicted. Both males and females were judged as more attractive when presented in photographs alongside an opposite-sex other. This finding is consistent with previous work showing ‘mate choice copying-like’ effects in men but is not consistent with findings in women [31, 40, 51]. Our findings suggest that sex differences in the effects of social context (including mate choice copying) on attractiveness may not be as strong as previously thought [21, 22, 27, 31, 38]. Interestingly, Little et al [43], reported that mate choice copying effects in men only occurred when the female model presented alongside the male ‘other’ was highly attractive. It is possible that the differences between our results and other studies that reported no mate choice copying effects on female attractiveness [31, 40] may be due to of systematic differences in the attractiveness or other properties of the ‘other’ men used.

Our finding that opposite sex ‘others’ affect both male and female attractiveness opens up the possibility that mate choice copying effects are not as sex-specific as previously thought, and it would be interesting to further investigate the sex-specificity of contextual effects and whether they lead to more profound consequences such as sex-specific cultural transmission of mate preferences [23, 52]. Further, individual differences regarding competitiveness and proclivity to copy the mate choices of others would be worthwhile investigating.

We predicted, from evolutionary Parental Investment Theory that there would be sex differences in judgments of mate-relevant traits. However, we found that ratings of male and female attractiveness were more similar than we predicted. Younger models of both sexes were rated as more attractive than older models and whilst our results for female models corroborate the established position cues of feminine youthfulness are attractive [5, 6], they directly oppose the common assertion that women, by preferring age-dependent traits such as social status and wealth, should find older men physically more attractive than younger men [7, 8]. It must be noted, however, that because this study was conducted online, the majority of the participants come from Western, Educated, Industrialised, Rich and Democratic (WEIRD) society [53]. In order to gain a more complete understanding of mate choice copying and the sex differences therein, tests conducted outside of a WEIRD demographic would be valuable.

In contrast to attractiveness ratings, the effects of social context on social status (rated earnings) showed more pronounced differences between the sexes. Male social status ratings were highest when males were presented alongside another male, whereas female social status was highest when presented alone, and significantly lower when in the presence of a male. We also found no evidence of a ‘beauty premium’, as younger females were rated as more attractive but not higher earning. Sex differences in status and in incentives to strive for status have complex, pervasive influences, not only on gender identity and behaviour, but also on patterns of economic activity [54]. Status seeking and alliance formation are associated with one another and with men’s reproductive success [55]. Further, high status men form same-sex alliances and partnerships [56]. In our study, the presence of two men not obviously in conflict may have given the appearance that the model male was adept at making same-sex partnerships, which could explain the higher ratings of male status within intra-sexual social contexts.

In wage-earning economies, men tend to earn more money, on average, than women. In the U.S and Australia, women’s median earnings are lower than men across the vast majority of fields [57–60]. Even in industries where more women are employed than men, median weekly earnings of men ($1058AUS) exceed those of women ($774AUS) [49, 50]. Our understanding of how economic gaps arise and persist has focused on technological factors that favour men [61, 62], sex role stereotyping [63], the effects of career breaks for maternity and career leave [64], and differences in male and female expected generosity [65], negotiating and networking styles [66].

Stereotypical or implicit assumptions that women earn less money than the men they work with are romantically involved with, or whose company they otherwise keep, can be both shaped by and feed back into sex-differences in earnings. In speed dating experiments, men value a woman’s intelligence and ambition only if it does not exceed their own levels of intellect and ambition [67]. Heterosexual relationships are more likely to dissolve when a woman’s monetary income approaches or exceeds that of her male partner [68, 69]. Relatively high earning women may engage in compensatory behaviour, such as leaving the workforce, reducing their hours, or over-expressing their femininity by taking up more household chores in order to preserve the relationship [70, 71]. Women’s earnings appear to be constrained by the amount that their male partners earn, in ways that do not apply when a man out-earns a woman. Our results suggest that even in a simplistic experimental exercise, participants are unwilling to assign economic status to a woman that exceeds that of the men with whom they are seen. This adds to the body of research suggesting that women who seek economic independence may face social exclusion [72, 73], and that economic inequality between men and women may be influenced by interactions between social status and attractiveness, presenting a persistent obstacle to the achievement of economic and social gender equality.

Our results suggest that our predictions derived from parental investment theory were too simplistic to be upheld. While PIT has made an enormous and important impact on the study of human mate choice and reproductive strategies, it has been criticised for focussing too tightly on the sex differences [74–76] and “male competition, female choice” paradigms at the expense of a more nuanced “Mutual Mate Choice” (MMC) understanding of human mate choice [3]. Recent studies, working within a MMC paradigm, have demonstrated how highly sensitive mate preferences can be to the context of an individual’s social and economic circumstances. For instance, Zentner and Mitura [77] demonstrated the importance of gender parity (in access to economic resources) on mate preferences; with men and women sharing more similar mate preferences and attitudes towards sex in societies with higher gender equality. Our study demonstrates how simple contextual cues can add considerable variability and nuance to judgements of attractiveness and social status. It also reveals asymmetries in the social constraints that apply to judgments of female and male social status. We hope our findings inspire further investigation into these asymmetries, and for the introduction of experimental evidence to discussions between biology, economics and sociology about sexism, its consequences, and policy design that might mitigate its effects.

## Supporting Information

S1 File Participant Information Sheet. Information about the study. This was presented to participants before they agreed to start the study.
(DOCX)

S2 File Excel spreadsheet containing data used in experiments. The spreadsheet contains the data used to perform the analysis.
(XLSX)

S1 Table MLMs for rated attractiveness of male and female models. The final MLMs of rated attractiveness of male and female models by men and women.
(DOCX)

S2 Table MLMs for rated earnings for target male and female participants. The final MLMs of rated earnings of male and female models by men and women.
(DOCX)
