# Supplementary material for: Sexual Conflict and Gender Gap Effects: Associations between Social Context and Sex on Rated Attractiveness and Economic Status
Source: PLoS One. 2016 Jan 5;11(1):e0146269. doi: 10.1371/journal.pone.0146269 (PMC4701490; doi:10.1371/journal.pone.0146269)

**PARTICIPANT INFORMATION**

**Before taking the study, you must agree to the following:**

**Title:** The Links between Mating, Status and Attractiveness**.**

**Participant Selection and Purpose of Study:** You are invited to participate in a study that explores the cues of status and attractiveness.

**Description of Study and Risks:** If you decide to participate, you will be asked to complete 3 tasks. This will take a total of 15 minutes, after which you will be fully debriefed and provided with contact details of the researchers should you wish to obtain further information about this project.

The tasks you will be asked to complete is:

1. We will ask you to provide demographic details: your age, sex, relationship status, ethnicity, educational and earning history.
2. You will then be asked to rate images of people. The images will be of natural people and the rating system will be explained to you prior to starting.
3. Finally, we will then ask you to complete the Kinsey Scale, which asks you to identify your predominant sexual orientation using a graded scoring system.

**Confidentiality and Disclosure of Information**: The information you provide will be given a unique code and will never be linked to your name. You will never be asked your name or any other identifying information.

**Your consent**: This study is entirely voluntary and you are free to withdraw yourself and any data that have been collected without prejudice at any point during the research. If you choose to withdraw at any point during the study, all the information gathered from you up to that point would be destroyed.

**Inquiries:** If you have any questions or concerns following your participation please contact Ms. Amany Gouda-Vossos (9385 8068) or Professor Rob Brooks (9385 2587).

Complaints may be directed to the Ethics Secretariat, The University of New South Wales, SYDNEY 2052 AUSTRALIA (phone 9385 4234, fax 9385 6648, email [ethics.sec@unsw.edu.au](mailto:ethics.sec@unsw.edu.au)).


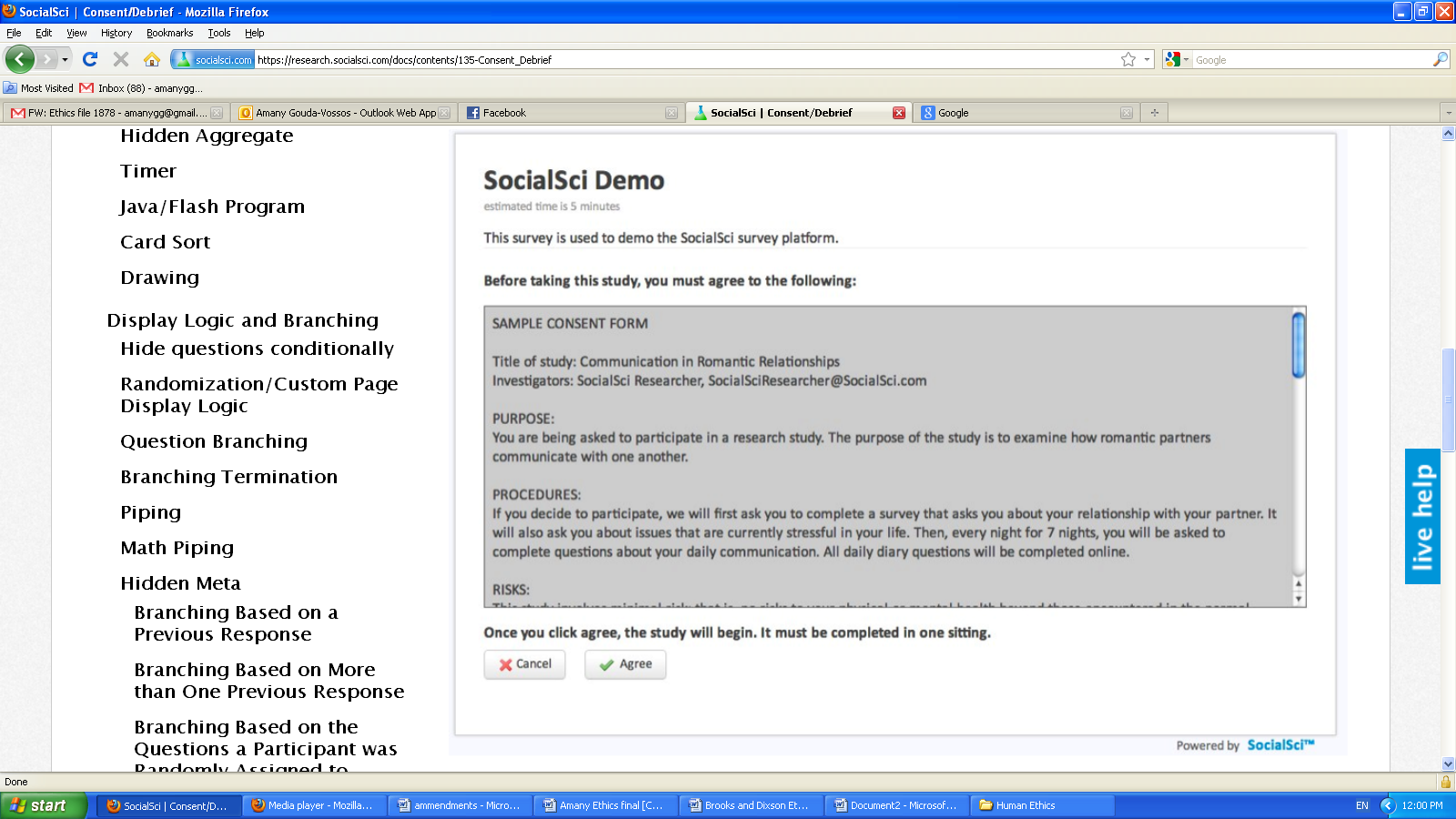

Supplement: S1 File — Information about the study. This was presented to participants before they agreed to start the study. (DOCX) [file pone.0146269.s001.docx]
