# Supplementary material for: Sexual Conflict and Gender Gap Effects: Associations between Social Context and Sex on Rated Attractiveness and Economic Status
Source: PLoS One. 2016 Jan 5;11(1):e0146269. doi: 10.1371/journal.pone.0146269 (PMC4701490; doi:10.1371/journal.pone.0146269)
Supplement: S1 Table — The final MLMs of rated attractiveness of male and female models by men and women. (DOCX) [file pone.0146269.s003.docx]

|  | **Target Male Attractiveness** | | |  | **Target Female Attractiveness** | | |
| --- | --- | --- | --- | --- | --- | --- | --- |
|  | ***d.f.*** | **F** | ***P*** |  | ***d.f.*** | **F** | ***P*** |
| **MLM 1 : Fixed Factors Only** |  |  |  |  |  |  |  |
| **Social Context** | 2,6605.2 | 6.043 | **0.002*** |  | 2,8619.3 | 15.968 | **<0.001*** |
| **Participant Sex** | 1, 6643.5 | 47.669 | **<0.001*** |  | 1, 8628.9 | 197.735 | **<0.001*** |
| **Social Context * Participant Sex** | 2, 6605.2 | 1.430 | 0.239 |  | 2, 8619.3 | 1.174 | 0.309 |
| **MLM 2: Including Covariate** |  |  |  |  |  |  |  |
| **Target Age** | 1, 3605.2 | 290.323 | **<0.001*** |  | 1, 4115.2 | 1831.295 | **<0.001*** |
| **Social Context * Target Age** | 2, 3590.6 | 2.903 | *0.055* |  | 2, 4108.2 | 12.129 | **<0.001*** |
| **Participant Sex * Target Age** | 1, 3605.2 | 14.375 | **<0.001*** |  | 1, 4115.2 | 174.517 | **<0.001*** |
| **Social Context * Participant Sex * Target Age** | 2, 3590.6 | 3.894 | **0.020*** |  | 2, 4108.2 | 1.766 | 0.171 |
