# Supplementary material for: Sexual Conflict and Gender Gap Effects: Associations between Social Context and Sex on Rated Attractiveness and Economic Status
Source: PLoS One. 2016 Jan 5;11(1):e0146269. doi: 10.1371/journal.pone.0146269 (PMC4701490; doi:10.1371/journal.pone.0146269)
Supplement: S2 Table — The final MLMs of rated earnings of male and female models by men and women. (DOCX) [file pone.0146269.s004.docx]

|  | **Target Male Earnings** | | |  | **Target Female Earnings** | | |
| --- | --- | --- | --- | --- | --- | --- | --- |
|  | ***d.f.*** | **F** | ***P*** |  | ***d.f*** | **F** | ***P*** |
| **MLM1: Fixed Factors Only** |  |  |  |  |  |  |  |
| **Social Context** | 2,5473.9 | 9.000 | **<0.001*** |  | 2,7972.2 | 80.182 | **<0.001*** |
| **Participant Sex** | 1, 5529.8 | 7.825 | **0.005*** |  | 1, 7980.5 | 0.966 | 0.326 |
| **Social Context * Sex** | 2, 5473.9 | 1.080 | 0.340 |  | 2, 7972.2 | 1.604 | 0.201 |
| **MLM 2: Including Covariates** |  |  |  |  |  |  |  |
| **Target Age** | 1, 2745.9 | 1439.191 | **<0.001*** |  | 1, 6103.6 | 288.596 | **<0.001*** |
| **Target Age2** | 1,3030.0 | 980.531 | **<0.001*** |  | 1, 6307.3 | 207.468 | **<0.001*** |
| **Social Context * Target Age** | 2, 2745.4 | 5.456 | **0.004*** |  | 2, 6099.9 | 11.891 | **<0.001*** |
| **Social Context * Target Age2** | 2, 3028.8 | 5.054 | **0.006*** |  | 2, 6302.9 | 12.739 | **<0.001*** |
| **Participant Sex * Target Age** | - | - | - |  | 1, 6103.6 | 0.026 | 0.873 |
| **Participant Sex * Target Age2** | - | - | - |  | 1, 6307.3 | 0.103 | 0.748 |
| **Social Context * Participant Sex * Target Age** | - | - | - |  | 2, 6099.9 | 5.173 | **0.006*** |
| **Social Context * Participant Sex * Target Age2** | - | - | - |  | 2, 6302.9 | 5.069 | **0.006*** |
